# Nitrogen metabolism in mycobacteria: the key genes and targeted antimicrobials

**DOI:** 10.3389/fmicb.2023.1149041

**Published:** 2023-05-18

**Authors:** Yufan Xu, Shiwei Ma, Zixin Huang, Longlong Wang, Sayed Haidar Abbas Raza, Zhe Wang

**Affiliations:** ^1^Shanghai Collaborative Innovation Center of Agri-Seeds, School of Agriculture and Biology, Shanghai Jiao Tong University, Shanghai, China; ^2^Shanghai Key Laboratory of Veterinary Biotechnology, School of Agriculture and Biology, Shanghai Jiao Tong University, Shanghai, China; ^3^Guangdong Provincial Key Laboratory of Food Quality and Safety/Nation-Local Joint Engineering Research Center for Machining and Safety of Livestock and Poultry Products, South China Agricultural University, Guangzhou, China

**Keywords:** nitrogen metabolism, antimicrobials, drug targets, tuberculosis, TB

## Abstract

Nitrogen metabolism is an important physiological process that affects the survival and virulence of *Mycobacterium tuberculosis*. *M. tuberculosis*’s utilization of nitrogen in the environment and its adaptation to the harsh environment of acid and low oxygen in macrophages are closely related to nitrogen metabolism. In addition, the dormancy state and drug resistance of *M. tuberculosis* are closely related to nitrogen metabolism. Although nitrogen metabolism is so important, limited research was performed on nitrogen metabolism as compared with carbon metabolism. *M. tuberculosis* can use a variety of inorganic or organic nitrogen sources, including ammonium salts, nitrate, glutamine, asparagine, etc. In these metabolic pathways, some enzymes encoded by key genes, such as GlnA1, AnsP2, etc, play important regulatory roles in the pathogenesis of TB. Although various small molecule inhibitors and drugs have been developed for different nitrogen metabolism processes, however, long-term validation is needed before their practical application. Most importantly, with the emergence of multidrug-resistant strains, eradication, and control of *M. tuberculosis* will still be very challenging.

## 1. Introduction

The first recorded case of tuberculosis (TB) can be traced back to 9000 years ago in the Eastern Mediterranean (Holzheimer et al., 2021). Human and zoonotic TB is normally caused by the infection of *Mycobacterium tuberculosis complex* which includes several species. They share highly conserved genomic sequences and possibly evolved from a single ancestor. During the past 200 years, TB kills more than 1 billion people (Gagneux, 2018). Furthermore, TB can also co-infected with other disease, leading to more serious symptoms (Huang and Zhao, 2022). As a matter of fact, co-infection with *Mycobacterium tuberculosis* (*M. tuberculosis*), becomes the primary reason of death to HIV-1 infectors. Despite some progress in diagnosis in recent years (Liao et al., 2022), the situation is still not promising. The mortality increased to 423,000 in 2020 (from 209,000 in 2019) (Bell and Noursadeghi, 2018; Harding, 2020), suggests that high rates of HIV infection have more TB cases and higher mortality rates (Dean et al., 2022). This increase shows that TB poses a significant threat to people’s lives whether they are co-infected with HIV or not.

In 2020, *M. tuberculosis*, the etiological agent of TB, caused 5.8 million new cases around the world and there may be 4.1 million people who haven’t been diagnosed or reported as WHO estimated (Harding, 2020). Despite the enormous financial and human resources invested in combating this deadly disease, they remain inadequate. Targets set in 2015 to reduce the number of infections and deaths have both not been met, with only half (11–20%) and a quarter (9.2–35%) of the plans achieved, respectively (Kirby, 2021). Especially with the COVID-19 pandemic, massive medical resources have been taken up, suggesting that we are facing a great hardship even more difficult to overcome than precedented. More worrying is that some studies suggest that the immune suppression and storm of inflammatory cytokines caused by COVID-19 may contribute to the development of TB (Hildebrand et al., 2022). The recent emergence of a case of latent TB reactivation due to COVID-19 infection (Leonso et al., 2022) has heightened this concern, as the proposed causes were the immunosuppressive sequelae of COVID-19, the effects of the chemotherapy (steroids and remdesivir), or a combination of both. Given the large numbers of people infected by both diseases, we must be alert to the potentially serious consequences of co-infection.

As a kind of facultative intercellular pathogen, *M. tuberculosis* can multiply in macrophages despite the acidic environment rich in radicals, and lacking in oxygen and nutrition, which usually kills most other bacteria (Höner zu Bentrup and Russell, 2001). In the dilemma, *M. tuberculosis* reduces metabolic activity and enters a dormant state which increases its resistance. However, most antibiotics target the processes of DNA replication, translation, or cell wall formation which are active in rapidly dividing cells. This makes it really hard to treat the infection. Patients are advised to use 4 drugs combination including isoniazid, rifampin, ethambutol, and pyrazinamide for 2–6 months and the therapy for multidrug-resistant cases even costs more time, 9–20 months (Caño-Muñiz et al., 2018). Even after a long period of treatment, if a small percentage of the pathogen is not killed, the probability of recurrence is very high. Therefore, exploring how *M. tuberculosis* survives in macrophages is really important and it’s attractive to develop novel drugs based on these studies.

There have been lots of studies on carbon metabolism reported in the literature, which have shown various carbon sources that *M. tuberculosis* used, the ways the bacterium obtained them from hosts, and specific metabolic pathways that it exploited during infection (Ehrt et al., 2018). By contrast, studies on the metabolism of nitrogen, another equally important element involved in the synthesis of many biomolecules including amino acids, proteins, nucleotides, some cofactors, and peptidoglycan, in this pathogen have just begun. A great deal of mystery remains about the processes of acquiring and assimilating nitrogen. In this review, we describe some of the key genes identified so far in the regulation of nitrogen metabolism, as well as some small molecule inhibitors.

## 2. Key genes in nitrogen metabolism

**FIGURE 1:**
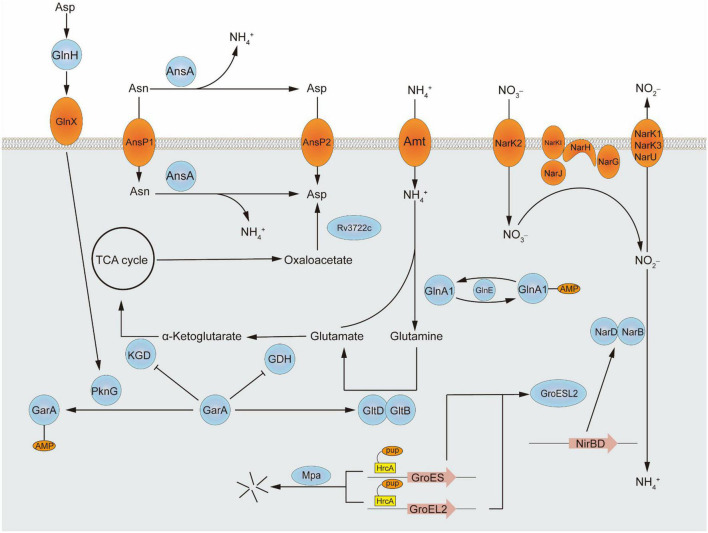
The sketch of nitrogen metabolism pathway in mycobacteria.

**FIGURE 2:**
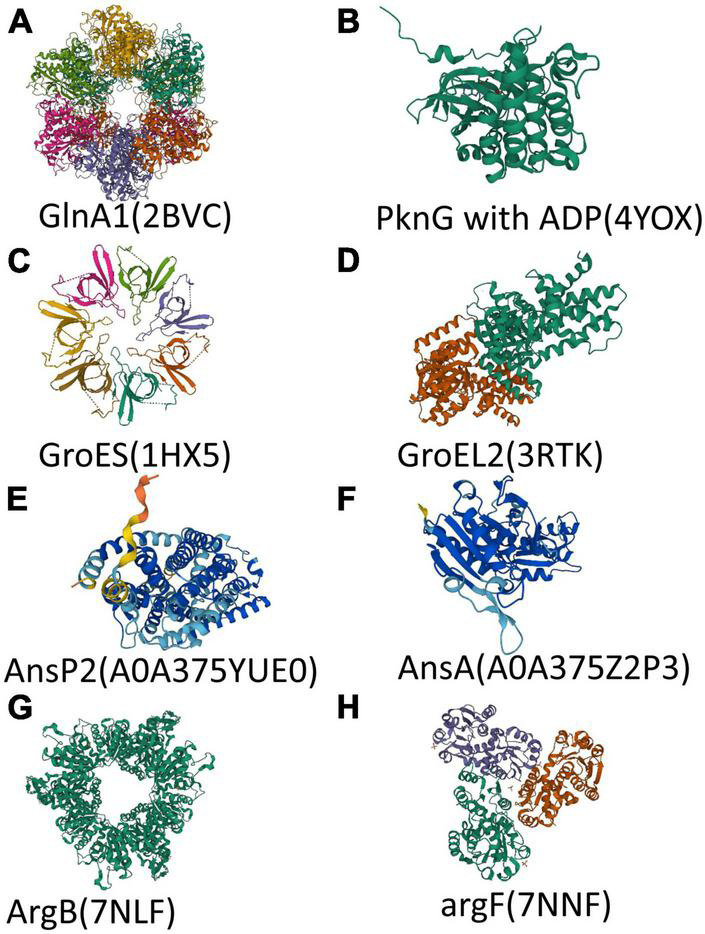
Structure of polypeptide chains encoded by key genes. **(A–H)** Overall structure of protein or polypeptide chain encoded by *glnA1*, *pknG*, *groES*, *groEL2*, *ansP2*, *ansA*, *argB*, and *argF*. The 3D model of AnsP2 and AnsA came from AlphaFold protein structure database (https://alphafold.com). Different colors in a 3D model represent per-residue confidence score (pLDDT) between 0 and 100 (Dark blue: pLDDT > 90, Light blue: 90 > pLDDT > 70, Yellow: 70 > pLDDT > 50, Orange: pLDDT < 50). The 3D model of other proteins came from RCSB PDB (https://www.rcsb.org/).

Central nitrogen metabolism, the best-studied part of nitrogen metabolism so far, concerned with the intake and utilization of ammonium, is indispensable to all living organisms around the world to balance internal and adapt to the external environment. *In vivo*, ammonium assimilation metabolism can be divided into two kinds according to the different conditions of glutamic acid synthesis. One is the formation of glutamate from ammonium and α- ketoglutaric acid in response to glutamate dehydrogenase and the other is a molecule of glutamine and a molecule of α-ketoglutarate catalyzed by glutamate synthetase to form two glutamates. The former has a lower Km value and is therefore referred to as a low-affinity pathway, while the latter is referred to as a high-affinity pathway. The major assimilation pathway in *M. tuberculosis* is the high-affinity pathway related to glutamine synthetase and glutamine oxoglutarate aminotransferase (GOGAT), which catalyze the production of glutamine or glutamate, respectively. Although a low-affinity pathway for ammonium assimilation using glutamate dehydrogenase exists in other bacteria, in *M. tuberculosis* the enzyme acts primarily on glutamine catabolism which breaks down glutamine into α-ketoglutaric acid and ammonium. Besides ammonium, the source of nitrogen can be various either inorganic substances such as nitrate or organics including alanine, aspartate, asparagine, glutamate, and glutamine (Agapova et al., 2019). Furthermore, nitrogen from different amino acids goes to different places. For example, according to the results of the isotope tracer, more than 50% of ^15^N from ^15^N1-Asp was transferred to Glu/n, ^15^N1-Glu to six amino acids, ^15^N2-Gln to eight amino acids, and ^15^N1-Leu to Ile. Nitrogen from glutamine is the major source and can turn into many other amino acids. While alanine is used directly as a component of thallus rather than transformed (Borah et al., 2019). Interestingly, *M. tuberculosis* grows faster when utilizes organic nitrogen sources. Some of the metabolic pathways involved in this review are shown in Figure 1 and the structures of proteins encoded by key genes are shown in Figure 2.

### 2.1. glnA1

Glutamine and glutamate are two molecules that play irreplaceable roles in central nitrogen metabolism. When ammonium in the medium is used as primary nitrogen donors by the bacterium, it must combine with glutamine and glutamate first (Leigh and Dodsworth, 2007). And because of lacking of a low-affinity pathway for ammonium assimilation, *M. tuberculosis* cannot assimilate ammonia directly into glutamate. Thus glutamine synthetase and glutamate synthetase are the sole means of ammonia assimilation (Tullius et al., 2003). There are 4 kinds of isoforms of glutamine synthetase, GlA1, GlnA2, GlnA3, and GlnA4. Among these, GlnA1, GlnA3, and GlnA4 synthesize L-glutamine, whereas GlnA2 synthesizes the D-glutamine and D-isoglutamine required for cell wall biosynthesis. However, only *glnA1* expresses abundantly, the three other genes being 9–15-fold less expressed (Harth et al., 2005). Though *glnA3* will overexpress when GlnA1 is inhibited, the quantity is less than fold which is too little to compensate for the lack of *glnA1* (Lee et al., 2006; Carroll et al., 2011), thus GlnA1 provides the main activity in *M. tuberculosis*. The gene *glnA1* consists of 1437 base pairs and encodes 478 amino acids (57.3 kDa) (Couturier et al., 2015). In addition to its internal role in bacteria, GlnA1 can also be secreted into the external environment as a secreted protein, which may affect phagosomal pH and phagosomal lysosomal fusion, thus allowing *M. tuberculosis* to survive phagocytosis by macrophages (Harth et al., 1994). What’s more, as reported, GlnA1 is related to the synthesis of poly-l-glutamate/glutamine. This component, which is absent in non-pathogenic mycobacteria, corresponds to 10% of the cell wall of pathogenic mycobacteria (Couturier et al., 2015). These all imply the importance of the gene *glnA1*.

In other bacteria represented by *E. coli*, the regulation of GlnA1 is a cascade reaction, mainly dependent on three proteins, PI protein, PII protein, and Gln D. Among them, PI has adenylyl transferase activity, which can adenylate or deadenylate GlnA1, thereby regulating its activity. There are two forms of PII: PII-UMP and defridulated. PII can promote PI-catalyzed adenylation, while PI-UMP can promote deadenylation. Different forms of PII are regulated by the uridylyl transferase GlnD (Read et al., 2007). In *M. tuberculosis*, the homologous proteins of the three proteins are GlnE, GlnK, and GlnD. But there are some differences between its function and that of *E. coli*.

According to many studies, both GlnA1 and GlnE are necessary for the growth of *M. tuberculosis*. This is specific to *M. tuberculosis*, studies have shown that GlnE is not necessary for *E. coli* and *Streptomyces* (Fink et al., 1999). GlnE has both adenylation and deadenylation activities, which regulate the activity of glutamine synthetase by transferring AMP to, or removing AMP from GlnA1. When GlnA1 binds to AMP, it becomes inactive. But studies that deleted adenylation or deadenylation domains separately showed that only the former was necessary for growth, and that the absence of the latter had no significant effect on growth (Carroll et al., 2008). This may be because if all the highly expressed GlnA1 is in the active state, the intracellular glutamate and ATP will be rapidly depleted. Given that glutamate is required for the sole pathway for *M. tuberculosis* to assimilate external ammonium, this could explain the lethality of the mutation.

In addition, GlnD showed adenylyl transferase but not uridylyl transferase activity in *M. tuberculosis*, controlling GlnK adenylation or deadenylation (Williams et al., 2013). Unlike *E. coli*, GlnK and GlnA1 adenylation appear to be independent of each other in *M. tuberculosis*. Studies have shown that *glnD-null mutants* do not show growth defects, although the expression of *amt*, *glnD*, and *glnK* genes is upregulated under nitrogen deficiency conditions (Read et al., 2007). Therefore, the role of GlnK and GlnD in *M. tuberculosis* nitrogen metabolism needs to be further studied.

Furthermore, it is also reported that GlnA1 has the activity of acyltransferase in active sites different from typical theories (Baghel et al., 2011). It means that glnA1 may not only regulate nitrogen metabolism by controlling the use of sources but also by modulating the activity of other proteins while being regulated by GlnE.

### 2.2. pknG

The GOGAT of *M. tuberculosis* is composed of two subunits, GltB and GltD (Cole et al., 1998), which play a very important role in nitrogen metabolism and adaptation to adverse environments. Some studies have shown that GltB/D catalyzed reactions can be used to neutralize cytoplasmic pH that is acidified while consuming host propionate carbon through the methylcitrate cycle. In contrast, there are defects in *M. bovis* BCG, which is not pathogenic and is less adaptable (Lee et al., 2018). Prior to this, it has been reported that the loss of *gltB* or *gltD* in *M. bovis* BCG would cause glutamatergic nutrient deficiency, resulting in inadequate growth of the strain in a medium with glutamate as the sole nitrogen source (Viljoen et al., 2013). These all implied that *gltB* and *gltD* may be related to the pathogenicity of *M. tuberculosis*.

Similar to GlnA1 above, GOGAT activity is regulated by a series of reactions. The glycogen accumulation regulator A (GarA) plays a regulating role in GOGAT and GDH. On the one hand, it can activate GOGAT to promote glutamate synthesis, on the other hand, it can inhibit GDH to inhibit glutamate decomposition. When GarA is phosphorylated, it becomes inactive, and metabolism shifts to glutaminolysis. The interconversion between glutamate and α-ketoglutarate can be regulated by phosphorylation and the dephosphorylation of GarA.

Further studies revealed that GarA phosphorylation and dephosphorylation were regulated by Serine-threonine protein kinase PknG (O’Hare et al., 2008). Disruption of *pknG* or *garA* has opposite effects on metabolism: defects in glutamate catabolism or intracellular glutamate depletion, respectively. Interestingly, disruption of GarA phosphorylation sites caused the same defects as *pknG* depletion (Rieck et al., 2017). In conclusion, a slight change in *pknG* expression can sensitively regulate metabolism through cascade amplification.

According to the crystal structure, there is no direct amino acid binding site for glutamine on PknG, so its activity needs the help of auxiliary components (Scherr et al., 2007). PknG consists of three distinct structural domains: rubredoxin domain (RD), kinase domain (KD), and tetratricopeptide repeat-containing domain (TPRD). The KD is sandwiched between RD and TPRD (Lisa et al., 2015). Some studies have shown that the activity of PknG is related to the environment outside the bacteria. The conserved aspartate-specific solute binding protein GlnH links extracellular amino acid concentration to PknG activity via the periplasmic transmembrane protein GlnX. There is a specific binding site for aspartic acid on GlnH. When amino acids are abundant in the environment, the conformation of GlnH bound to aspartic acid will be changed and bind to the transmembrane protein GlnX, and the signal will be transmitted to the cell, causing the activation of PknG. Activated PknG phosphorylates and inactivates GarA, thereby regulating glutamate metabolism (Bhattacharyya et al., 2018). In addition, studies of PnkG substrates by affinity purification-mass spectrometry revealed that PknG may regulate many physiological processes, such as nitrogen and energy metabolism, cell wall synthesis, and protein translation (Gil et al., 2019). Metabolomic analysis showed that PknG was necessary to adapt to hypoxia and maintain REDOX balance, and was associated with a dormancy state (Khan et al., 2017).

### 2.3. groES and groEL2

In addition to ammonia, nitrate is also an important source of nitrogen available to *M. tuberculosis*. It was a few decades ago, researchers have found that nitrate can be the only nitrogen source to support the growth of *M. tuberculosis* (Hedgecock and Costello, 1962). Nitrate is also essential for the survival of *M. tuberculosis* under hypoxic conditions. It can act as a terminal electron acceptor in the respiratory chain under hypoxic conditions, causing *M. tuberculosis* to enter a dormant state instead of dying (Aly et al., 2006; Via et al., 2008). In addition, nitrite produced by reduction during nitrate metabolism can protect bacteria from the damage of reactive oxygen species and reactive nitrogen species *in vivo*. The metabolic pathway of nitrate is quite conservative. It is reduced to nitrite, which is further reduced to ammonia by NirBD, and then enters the central nitrogen metabolism. The nitrite that is not immediately reduced during this process is expelled from the cell (Malm et al., 2009).

Recent studies have found that the reduction of nitrate by *M. tuberculosis* requires chaperones expressed by *groES* and *groEL2*. They’re all heat shock proteins (HSP) due to their increased levels of expression at elevated temperatures. As chaperone proteins, HSPs are widely and highly conserved in various organisms. They perform ATP-dependent folding of proteins to ensure cell viability at different temperatures (Ellis, 1999). The most characterized one among them is the GroEL/ES complex, or called Cpn60/Cpn10, from *E. coli*. GroEL forms two heptamer rings, which are then stacked back-to-back to form a complex of tetradecamers. GroES, on the other hand, bind to one or both ends of the GroEL complex after forming a heptamer ring driven by ATP. Both Cpns are required for *E. coli* (Xu et al., 1997; Grallert and Buchner, 2001). But in *M. tuberculosis*, the situation is different. Although there are also homologous GroEL and GroES co-expressed by *groE* operon, there is another HSP in *M. tuberculosis* that belongs to the HSP60 family, commonly referred to as GroEL2. The gene that encodes the protein is not on *groE* operon (Qamra et al., 2005). And experiments showed that when the genes encoding GroES or GroEL2 were deleted, *M. tuberculosis* growth was completely inhibited. However, the lack of GroEL only prevented granulomatous inflammation in infected mice or guinea pigs (Hu et al., 2008). In addition, unlike *E. coli*, neither GroEL nor GroEL2 forms tetradecamers in *M. tuberculosis* cells, but exists as oligomers (Qamra et al., 2004). Crystal structures show that GroEL2 forms dimers in *M. tuberculosis* (Qamra and Mande, 2004). Furthermore, it has been proposed that GroEL2 has only weak ATPase activity and can function in an ATP-independent mode (Qamra et al., 2004). This may be due to the slower growth of mycobacteria, which helps it survive in harsh environments such as hypoxia and poor nutrition. The HrcA acts as a transcriptional repressor to block the expression of *groES* and *groEL2* though binding to their cognate DNA elements. Together with the pup-proteasome system, they regulate nitrate utilization. When the pup-proteasome system is absent and HrcA cannot be degraded, *M. tuberculosis* is deficient in nitrate utilization (Becker et al., 2019). Furthermore, the GroESL2 complex may also be able to fold other proteins involved in nitrogen metabolism. Researchers can explore the role of this regulatory mechanism in other pathways and there may be more discoveries.

### 2.4. ansP2, ansA, and Rv3722c

Asparagine is also important or even known as one of the best nitrogen sources for *M. tuberculosis*. There should be systems in place to transport and utilize this amino acid. It has been previously reported that *M. tuberculosis* can capture aspartate through membrane transporters and use the amino as a source of nitrogen during infection. Interestingly, AnsP2 (Rv0346c), the homolog of AnsP1 whose expression is markedly induced in the lungs of patients (Rachman et al., 2006), was recently predicted to be an asparagine transporter. However, although the growth of the knockout strain was affected when asparagine was used as the sole nitrogen source, the pathogenicity to mice was not reduced (Gouzy et al., 2014), suggesting that there may be other unknown transporters waiting to be discovered.

There are also some new achievements in the study of asparaginase. This enzyme was discovered decades ago in various mycobacteria, including *M. tuberculosis* (Kirchheimer and Whittaker, 1954). The AsnA/Rv1358c was previously found to hydrolyze aspartate *in vitro* in *Mycobacterium bovis* (Cai et al., 2012), and its homolog in *M. tuberculosis* was recently found to perform the same function. The activity of AsnA/Rv1358c has been reported and annotated, indicating that Rv1358c, as a secretory protein, can help *M. tuberculosis* resist an acidic environment by hydrolyzing asparagine to aspartic acid and ammonia. In addition, it is also recognized to induce stress to primary immune cells and compromise the host immune response (Gouzy et al., 2014; Kataria et al., 2021). Compared with central nitrogen metabolism, the research in this area is less and still in its infancy. Studying the function of genes involved in this metabolic pathway is an alternative research direction in the future.

In addition, as mentioned above, Glu is the primary portal of nitrogen assimilation and 27% of nitrogen is distributed via Asp for the dedicated biosynthesis of several cofactors, nucleotides, and amino acids (Reitzer, 2004). It highlights the importance of aspartate aminotransferase which connects metabolisms of glutamate and aspartate. Gratifyingly, *Rv3722c* was found to encode the main aspartate aminotransferase in *M. tuberculosis* (Jansen et al., 2020). What’s more, *Rv3722c* belongs to a recently described and structurally distinct subclass of AspATs, designated type Ic, whose members are absent in humans and almost exclusively present in bacteria (Son and Kim, 2016; El-Gebali et al., 2019). It lays an avenue between carbon and nitrogen metabolisms.

### 2.5. Gene of arginine synthesis

*Mycobacterium tuberculosis* preserves most of the essential nutrient synthesis pathways, allowing it to survive in a variety of nutrient-deficient environments, which may be one of the important reasons for its success. In previous studies, *M. tuberculosis* strains deficient in leucine or glutamate showed reduced growth but did not die (Hondalus et al., 2000; Lee et al., 2006). Only the absence of methionine or arginine causes bactericidal effects (Berney et al., 2015). Strains with knockdown of *argB* or *argF*, coding two key enzymes in the *de novo* arginine synthesis pathway, are unable to grow in an arginine-deficient medium. Of particular interest, although *M. tuberculosis* has two carriers for arginine transport and sufficient amino acids in host serum, this cannot compensate for the absence of *de novo* arginine synthesis (Tiwari et al., 2018). This was reflected in the experimental results that when *argB*-or *argF*-*deficient M. tuberculosis* was injected into mice, the mutants failed to replicate. It has previously been reported that *Mycobacterium bovis* cannot utilize *in vitro* arginine as the sole nitrogen source (Peteroy-Kelly et al., 2003). Given the similarity between the two bacteria, this may imply that *M. tuberculosis* is unable to utilize or only inefficiently transport exogenous arginine. In addition, oxidative damage caused by ROS accumulation was observed in *argB* or *argF null mutants*, which is the bactericidal mechanism of the commonly used anti-tuberculosis drug isoniazid (Middlebrook et al., 1954; Dhandayuthapani et al., 1996), suggesting the research direction of new bactericidal or adjuvant drugs. And since there is no metabolic pathway for *de novo* arginine synthesis in the body, it might be a target with fewer side effects.

## 3. Inhibitors of nitrogen metabolism

**FIGURE 3:**
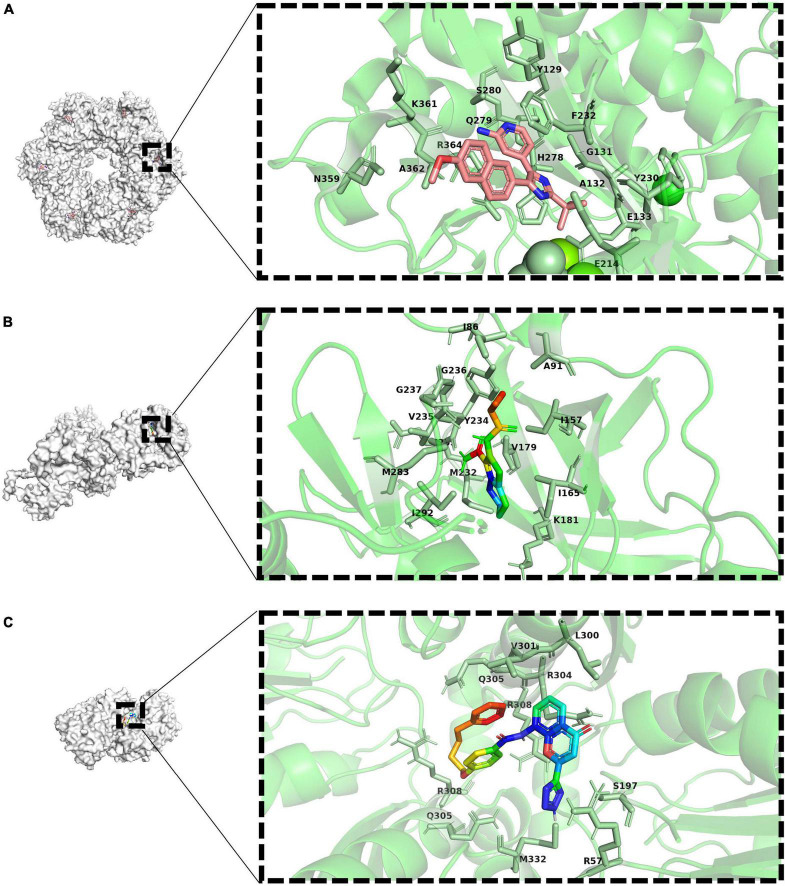
Docking view or co-crystal complexes of ligands and proteins. **(A)** Co-crystal complexe of 4-(2-Tert-Butyl-4-(6-Methoxynaphthalene-2-Yl)- 3 h-Imidazole-4-Yl) pyridine-2-Amine (PubChem-ID: 673481) as ligand and GlnA1 (Co-crystallization PDB-D: 3ZXV). Sticks representation of residues with 4Å around ligand in a zoomed view. **(B)** Co-crystal complexe of ax20017 (PubChem-ID:673481) as ligand and PknG (Co-crystallization PDB-D: 2PZI). Sticks representation of residues with 4Å around ligand in a zoomed view. **(C)** Docking view of Pranlukast (PubChem-ID:4887) as ligand and ArgJ (PDB-ID: 3IT4). Sticks representation of residues with 4Å around ligand in a zoomed view.

After long-term studies, a variety of anti-tuberculosis drugs are being used in clinical practice. For drug-sensitive *M. tuberculosis*, the combination of several common drugs, such as rifampicin, isoniazid, pyrazinamide and ethambutol, can achieve good results. But because of the existence of the dormant state, many patients suffer from a long course of treatment and easy to relapse. In addition, the large number of multi-drug resistant bacteria also put forward an urgent need for the development of new drugs. The inhibitors involved and their dosages are shown in Table 1 and the docking view of ligands and proteins is shown in Figure 3.

**TABLE 1:** Chemical inhibitors targeted to nitrogen metabolism.

Name	Structural formula	IC50	MIC	Target	References	PubChem ID
L-methionine-SR-sulfoximine	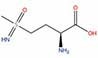	/	10 μM	Glutamine synthetase	Harth and Horwitz, 2003	801860
4-(2-Tert-Butyl-4-(6-Methoxynaphthalene-2-Yl)-3 h-Imidazole-4-Yl) pyridine-2-Amine	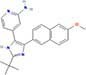	0.049 μM	2 μg/ml	Glutamine synthetase	Gising et al., 2012	56928064
AX20017	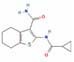	0.39 μM	/	PknG	Scherr et al., 2007	673481
Doxorubicin	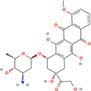	56 μM	/	Asparaginase	Kataria et al., 2019	31703
Pranlukast	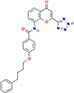	2.7 μg/ml	5.2 μg/ml	ArgJ	Mishra et al., 2019	4887

“/” Means the values are not mentioned in the references. The structure of L-methionine-SR-sulfoximine was drawn using Chemdraw and others came from PubChem database (https://pubchem.ncbi.nlm.nih.gov/).

### 3.1. Inhibitor of glutamine synthetase

As one of the most important enzymes in central nitrogen metabolism, glutamine synthetase is a natural candidate drug target. In addition, although GlnA1 is also present in humans, its homology with *M. tuberculosis* is low, less than 20% (Krajewski et al., 2008). Therefore, drugs developed with this target will have limited risks of on-target *in vivo* toxicity. Earlier drugs were mostly glutamine analogs that competitively bind glutamine synthetase. For example, L-methionine-Sr-sulfoximine (MSO) has been reported to reduce the number of *M. tuberculosis* in the lungs and spleen of diseased animals, and can synergize with isoniazid and ascorbic acid to improve the efficacy. As expected, MSO showed high selectivity for *M. tuberculosis* protein. The compound was 100-fold less active against human glutamine synthetase (Harth and Horwitz, 2003). Unfortunately, although the drug markedly reduced the amount of poly real–L–glutamate/glutamine cell wall structure in *M. tuberculosis*, the drug can’t cross the cell wall of mycobacteria. Therefore, only extracellular glutamine synthetase can be inhibited, but the effect on cellular glutamine synthetase is very small (Harth and Horwitz, 1999). For other mycobacteria, there is few report about the antibacterial efficacy of MSO. However, *in vitro* biochemical result shown that MSO can largely inhibit the enzymatic activity of glutamine synthetase in *Mycobacterium avium*, the representative strain of non-tuberculosis mycobacteria (NTM) (Alvarez and McCarthy, 1984).

Consider the process catalyzed by glutamine synthetase, ATP phosphorylates glutamate to form an intermediate, and then the amino group replaces the phosphate group to form glutamine, glutamine synthetase functions as an ATP-dependent process. Drugs that interact with ATP binding have been developed in recent years. Bedaquiline (BDQ), the first new drug approved in decades, is an inhibitor of *M. tuberculosis* ATP synthase. As expected, there has been some evidence supporting the synergy between MSO and BDQ (Wang et al., 2019). Several bisphosphonic acid derivatives have been reported to have potential as new drugs, and given their bone-targeting properties, these compounds are promising for the treatment of bone tuberculosis (Kosikowska et al., 2016). In addition, 4-(2-Tert-Butyl-4-(6-Methoxynaphthalen-2-Yl)-3 h-Imidazol-4-Yl) pyridin-2-Amine was also found to has antibacterial activity by high-throughput screening (Gising et al., 2012) (the drug-target interaction is shown as Figure 3A). However, it is highly toxic to human cells, so its specificity needs to be enhanced to develop its clinical use.

Recently, it has been reported that the anticancer drug linsitinib can act as a competitive inhibitor of ATP binding and affect GlnA activity, thereby inhibiting the growth of *M. tuberculosis*. It can also enhance the resistance to *M. tuberculosis* by activating autophagy in host cells. Similarly, it also showed a certain synergistic effect of bedaquiline (Wang et al., 2022). As a drug developed in the past, its safety is relatively guaranteed. However, its anti-tuberculosis effect needs to be strengthened and further optimized in subsequent studies.

These molecules with different roles and different sources provide a broad prospect for the development of new drugs.

### 3.2. Inhibitor of PknG

As mentioned in the previous content, PknG is related to many physiological processes in *M. tuberculosis*, participating in the regulation of various metabolism, and its metabolites connect carbon metabolism and nitrogen metabolism. In particular, it is associated with the survival and dormant state of *M. tuberculosis* under hypoxia. This is crucial for the survival of *M. tuberculosis* in macrophages and the development of drug resistance. It is well known that many TB drugs, such as isoniazid, mainly target bacteria in the replicating phase but have little effect on bacteria in the non-replicating dormant state. Therefore, PknG as a drug target may play a role in killing bacteria and weakening drug resistance, so as to achieve a better therapeutic effect.

Compound AX20017 was reported to occupy the binding site for ATP whose structure is out of the ordinary, inhibits PknG, and causes mycobacterial transfer into lysosomes, thereby killing mycobacteria (the drug-target interaction is shown as Figure 3B). Moreover, kinase-dependent processes within the macrophage host cell, such as the capacity to proliferate, synthesize proteins, and phagocytose do not interfered, so as the cellular morphology demonstrating that AX20017 is a highly specific inhibitor of PknG (Scherr et al., 2007). To improve the affinity, selectivity and potency of AX20017, the cyclopropyl ring which does not exploit binding capacity fully may be an alternative modification site. In recent years, although some studies have found RO9021 and a few flavonoids by computer virtual screening and simulation docking, no subsequent experiments based on cells or animals have been found (Qasaymeh et al., 2019; Arica-Sosa et al., 2022), so its effectiveness still needs to be verified and it is still far from clinical use. It is worth nothing that targeting PknG can also cause the growth inhibition of *M. bovis*, which has been proven through several independent reports (Singh et al., 2015; Kanehiro et al., 2018; Kidwai et al., 2019). The similar sequence of PknG among MTBC therefore let it become a promising broad-spectrum anti-mycobacteria drug target.

### 3.3. Inhibitor of asparaginase

As mentioned above, asparagine is one of the important nitrogen sources for *M. tuberculosis*. Asparaginase is mainly categorized into type I, II, and III. Type I and II are found in most bacteria, while type III, which has a distinct mode of catalysis is usually found in mammals and plants (Nomme et al., 2012). This difference makes the enzyme a good candidate as a drug target. Specific inhibitors targeting the active site of this enzyme, the potential inhibitors were predicted and screened from ZINC database, TCM database and FDA-approved drug database, according to the structure. Excitingly, M3 (ZINC 4740895), M26 (ZINC 33535) and doxorubicin were screened out and showed satisfactory results in *M. smegmatis* (Kataria et al., 2019). The experiment in *M. tuberculosis* has not been reported, which is worthy of further study. The result can also be extended to *Salmonella typhi*, *H. pylori*, and *L. donovan*, which encode significant overlaps in the conserved catalytic residues. This makes it possible that these compounds could be used as a basis for further development of broad-spectrum antimicrobial drugs to help patients with multiple bacterial co-infections. It was also reported that three kinds of phytocompounds, Physalin D, Withanone, and Withaferin A, can competitively bind asparaginase (Sharma et al., 2022). However, the effect on *M. tuberculosis* remains to be studied.

### 3.4. Inhibitor of arginine synthesis

Arginine is very important for the growth of *M. tuberculosis*, and the abundant arginine in the outside world cannot compensate for the influence caused by the blockage of its own synthetic pathway. In addition, there is no metabolic pathway for *de novo* arginine synthesis in human body. These make it possible to find drug targets in arginine anabolic metabolism. Most of the substrates for arginine synthesis are common small molecules, so if they are used as the targets of inhibitors, the drugs may have serious off-target effects. However, the allosteric sites of enzymes required for metabolism are less conserved in evolution, which can be used as drug-targeting sites to enhance specificity and reduce side effects (Wenthur et al., 2014). Recently, it has been reported that Pranlukast (PRK) can act as an allosteric inhibitor of ArgJ during arginine biosynthesis in *M. tuberculosis* and inhibit its ornithine acetyltransferase activity, thereby inhibiting bacterial survival and virulence (Mishra et al., 2019) (the drug-target interaction is predicted as shown in Figure 3C). Further metabolomics studies showed that PRK caused significant differential expression of 50 metabolites (Yelamanchi et al., 2022). This will contribute to a deeper understanding of PRK-mediated metabolic changes and provide a basis for the development of new therapeutic approaches. Because it has already been approved by the FDA, PRK could be ready for use much faster than a new drug. And as an approved drug, there are no excessive concerns about its safety, and patients are more likely to accept it.

## 4. Way forward

In this review, we describe some of the key genes involved in nitrogen metabolism in *M. tuberculosis* using ammonium and nitrate as inorganic nitrogen sources and some amino acids as organic nitrogen sources. Some inhibitors or drugs targeting nitrogen metabolism were also introduced. The action sites of the inhibitors are mainly some key enzymes in the metabolic process, which also indicates the direction of developing new anti-tuberculosis drugs in the future. Many new inhibitors have been discovered, giving hope for the development of new anti-TB drugs. However, many research results remain at the level of computer prediction or biochemical experiments, and the subsequent results of cell and animal experiments are lacking, which may be related to the greater risk of *Mycobacterium tuberculosis* and the higher requirements of laboratory safety level for research. Considering previous studies have reported some nanomolar inhibitions of GlnA1 inhibitors, none of which were active against whole-cell *M. tuberculosis* (Couturier et al., 2015), cautious optimism is warranted.

In addition, it is worth noting that some drugs not only act on the metabolic process of *M. tuberculosis*, but also kill the pathogen by affecting host cells. Recent studies have reported that mitochondrial metabolism regulated by mTOR limits mycobacteria-induced cytotoxicity (Pagán et al., 2022) suggesting that screening new anti-TB drugs from existing drugs that target mTOR may be a good direction. And compared with developing new drugs, existing drugs have great advantages in terms of safety and time to approval.

Like we have been through in the past, the clinical application of nitrogen-targeted antimicrobials will inevitably lead to drug resistance in the future. However, finding a synergistic drug combination includes 1, 2, or more extra antibiotics could be an effective way to overcome drug resistance. The previous research from our team has shown that inhibiting both ATP synthesis and glutamine synthesis causes a synergistic killing effect (Wang et al., 2019), it is therefore worth to keep exploring the combination between BDQ and other nitrogen metabolism targeted drugs, since ATP is the crucial factor driving hundreds of biochemical reactions to maintain the bacterial nitrogen homeostasis.

There are still many possible drug targets that have not been studied or rarely studied, leaving a wide range of possibilities for new drugs. However, due to the complexity of metabolic processes and the need to consider the effects of drugs on the patient’s body, the real development of effective and safe drugs will still be a long and arduous process.

## Author contributions

ZW and YX: conceptualization. YX, SM, and LW: literature search. ZH and YX: protein structure analysis. ZW, YX, and SM: writing—original draft preparation. ZW, YX, SM, LW, and SR: writing—review and editing. All authors read and agreed to the published version of the manuscript.
